# Prenatal Exposure to Intimate Partner Violence and Developmental Health in Children at Kindergarten: Linking Canadian Population-Level Administrative Data.

**DOI:** 10.23889/ijpds.v7i3.1860

**Published:** 2022-08-25

**Authors:** Janelle Boram Lee, Marni Brownell, Tracie Afifi, Lorna Turnbull, Marcelo Urquia, Nathan Nickel

**Affiliations:** 1 University of Calgary; 2 Manitoba Centre for Health Policy, University of Manitoba; 3 University of Manitoba

## Objectives

Using population-wide administrative data, the objective was to provide Canadian evidence on the longitudinal relationship between maternal intimate partner violence (IPV) victimization and children’s developmental health. Using provincial prosecution records, we examined developmental vulnerability (DV) at kindergarten of children prenatally exposed to maternal IPV victimization compared to unexposed counterparts.

## Approach

This retrospective cohort study linked administrative datasets (legal, health, education, social services) from the Population Research Data Repository at the Manitoba Centre for Health Policy. Exposed mother-child pairs with 1+ prosecution record of maternal IPV victimization during pregnancy between 2003 and 2018 in Manitoba (n = 1,117) were matched to unexposed pairs (1:3) based on sex/birthdate of child and neighbourhood income. DV at kindergarten was measured across 5 domains (physical, social, emotional, language/cognitive [LC], communication/general knowledge) using the Early Developmental Instrument (EDI). Children without eligible EDI scores were excluded. Multiple logistic regression models were conducted to address the objective.

## Results

The eligible cohort included 927 children (exposed n=229, unexposed n=698); 31.07% of the cohort was developmentally vulnerable in one or more domains (1/+) and 19.53% was developmentally vulnerable in two or more domains (2/+). Children who were prenatally exposed to maternal IPV victimization had increased odds of vulnerability across all 5 developmental domains (e.g., physical health/wellness: OR=2.83[1.95,4.10]; LC development: OR=2.45[1.65,3.64]). Unadjusted ORs showed statistically significant associations between maternal exposure of prenatal IPV victimization and DV in 1/+ (OR=2.70[1.98,3.68]) and 2/+ (OR=2.48[1.75,3.50]). When adjusted for covariates (e.g., maternal income assistance, mental health, child abuse history), no statistically significant relationship was found for any of the domains (e.g., LC development: aOR=0.98[0.53,1.81]), 1/+ (aOR=1.17[0.72,1.88]), and 2/+ (aOR=1.14[0.67,1.95]).

## Conclusion and Relevance

The unadjusted, statistically significant associations suggest children exposed to maternal IPV victimization prenatally may face associated social/health risks. The finding highlights the need to consider potential factors that put children at risk of DV when developing and implementing support systems/interventions for children exposed to maternal IPV victimization.

